# Dopamine D1 Receptor Gene Variation Modulates Opioid Dependence Risk by Affecting Transition to Addiction

**DOI:** 10.1371/journal.pone.0070805

**Published:** 2013-08-16

**Authors:** Feng Zhu, Chun-xia Yan, Yi-chong Wen, Jiayin Wang, Jinbo Bi, Ya-ling Zhao, Lai Wei, Cheng-ge Gao, Wei Jia, Sheng-bin Li

**Affiliations:** 1 College of Medicine and Forensics, Xi'an Jiaotong University Health Science Center, Xi'an, Shaanxi, People's Republic of China; 2 Key Laboratory of the Health Ministry for Forensic Sciences, Xi'an Jiaotong University, Xi'an, Shaanxi, People's Republic of China; 3 Key Laboratory of the Education Ministry for Environment and Genes Related to Diseases, Xi'an, Shaanxi, People's Republic of China; 4 The Genome Institute, Washington University, Saint Louis, Missouri, United States of America; 5 Department of Community Medicine and Health Care, School of Medicine University of Connecticut Health Center, Farmington, Connecticut, United States of America; 6 School of Public Health, Xi'an Jiaotong University Health Science Center, Xi'an, Shaanxi, People's Republic of China; 7 Department of Psychiatry, First Affiliated Hospital, Xi'an Jiaotong University Health Science Center, Xi'an, Shaanxi, People's Republic of China; 8 Methadone Maintenance Therapy Clinic, Xi'an Mental Health Center, Xi'an, Shaanxi, People's Republic of China; Yale University, United States of America

## Abstract

Dopamine D1 receptor (DRD1) modulates opioid reinforcement, reward, and opioid-induced neuroadaptation. We propose that *DRD1* polymorphism affects susceptibility to opioid dependence (OD), the efficiency of transition to OD, and opioid-induced pleasure response. We analyzed potential association between seven *DRD1* polymorphisms with the following traits: duration of transition from the first use to dependence (DTFUD), subjective pleasure responses to opioid on first use and post-dependence use, and OD risk in 425 Chinese with OD and 514 healthy controls. DTFUD and level of pleasure responses were examined using a semi-structured interview. The DTFUD of opioid addicts ranged from 5 days to 11 years. Most addicts (64.0%) reported non-comfortable response upon first opioid use, while after dependence, most addicts (53.0%) felt strong opioid-induced pleasure. Survival analysis revealed a correlation of prolonged DTFUD with the minor allele-carrying genotypes of *DRD1* rs4532 (hazard ratios (HR) = 0.694; *p* = 0.001) and rs686 (HR = 0.681, *p* = 0.0003). Binary logistic regression indicated that rs10063995 GT genotype (vs. GG+TT, OR = 0.261) could predict decreased pleasure response to first-time use and the minor alleles of rs686 (OR = 0.535) and rs4532 (OR = 0.537) could predict decreased post-dependence pleasure. Moreover, rs686 minor allele was associated with a decreased risk for rapid transition from initial use to dependence (DTFUD≤30 days; OR = 0.603) or post-dependence euphoria (OR = 0.603) relative to major allele. In conclusion, *DRD1* rs686 minor allele decreases the OD risk by prolonging the transition to dependence and attenuating opioid-induced pleasure in Chinese.

## Introduction

Opioid dependence (OD) is a complex disease influenced by both environmental and genetic factors [1]. Linkage and association studies have partially revealed the molecular basis of the heritability of OD [2]. However, many of the identified gene variations could not be replicated by independent studies [2]. The discrepancy could reflect genetic heterogeneity and/or minimal/moderate effects of any single gene. Distinct subtypes of the diagnosis with heterogeneous genetic determinants may have also contributed to the inconsistent observations [3], [4], [5]. Classification of opioid users into more homogeneous subgroups with clinical and/or pathophysiological features could help to identify involved genetic factors [5].

The duration for transition from first use to dependence (DTFUD) varies dramatically among addicts [6], and may determine addictive liability in particular subjects [7]. Animal experiments revealed varying vulnerability in transition to dependence [8] and an association of the transition to persistent impairment in synaptic plasticity [9]. Although several environmental factors have been reported to affect DTFUD [10], recent studies suggest that genetic factors play more important role in the transition to dependence [2], [11]. However, few susceptibility genes affecting the transition to dependence have been identified to date.

Euphoria induced by drugs of abuse is a critical drive for drug use and seeking [12]. The inter-individual variability in the strength of pleasure response on first heroin use has been attributed to μ-opioid receptor gene polymorphism [13]. The subjective pleasure response is a product of opioid rewarding property. Opioid rewarding effects can be intensified by repeated drug exposure [14], [15]. The pleasure response to opioid is more dependent on the drug-induced changes in brain reward circuitry after dependence than in early use. Relative to response to early use, the post-dependence response reflects more stably genetic vulnerability to reward dysregulation caused by opioid [16]. Reward processing depends on dopaminergic neurotransmission [17]. Previous studies indicated that genetic variations in dopaminergic pathway could affect the reward process, subjective response, and susceptibility to dependence [18].

Dopamine receptor D1 (*DRD1*) is a possible susceptibility gene for OD. A recent study found that a single nucleotide polymorphism (SNP) (rs265975) located at 5 kb downstream of *DRD1* is associated with opiate abuse in Caucasians [19]. *DRD1* polymorphism (rs4532, rs686, and rs265981) has also been associated with substance dependence [20], [21], addictive behavior [22], and psychiatric diseases [23], [24]. The rs686 polymorphism affects *DRD1* expression and may influence the DRD1 activation in prefrontal cortex [20], [25].

In the current study, we examined DTFUD and subjective response to opioid on first use and post-dependence use in 425 opioid addicts via retrospective investigation. We genotyped seven possibly functional SNPs in the *DRD1* regulatory and coding regions in these addicts and 514 healthy controls. We evaluated the relationships between *DRD1* polymorphisms and DTFUD, the response to opioid on first use, post-dependence, and risk for OD.

## Experimental Procedure

### 2.1 Samples

**Figure 1:**
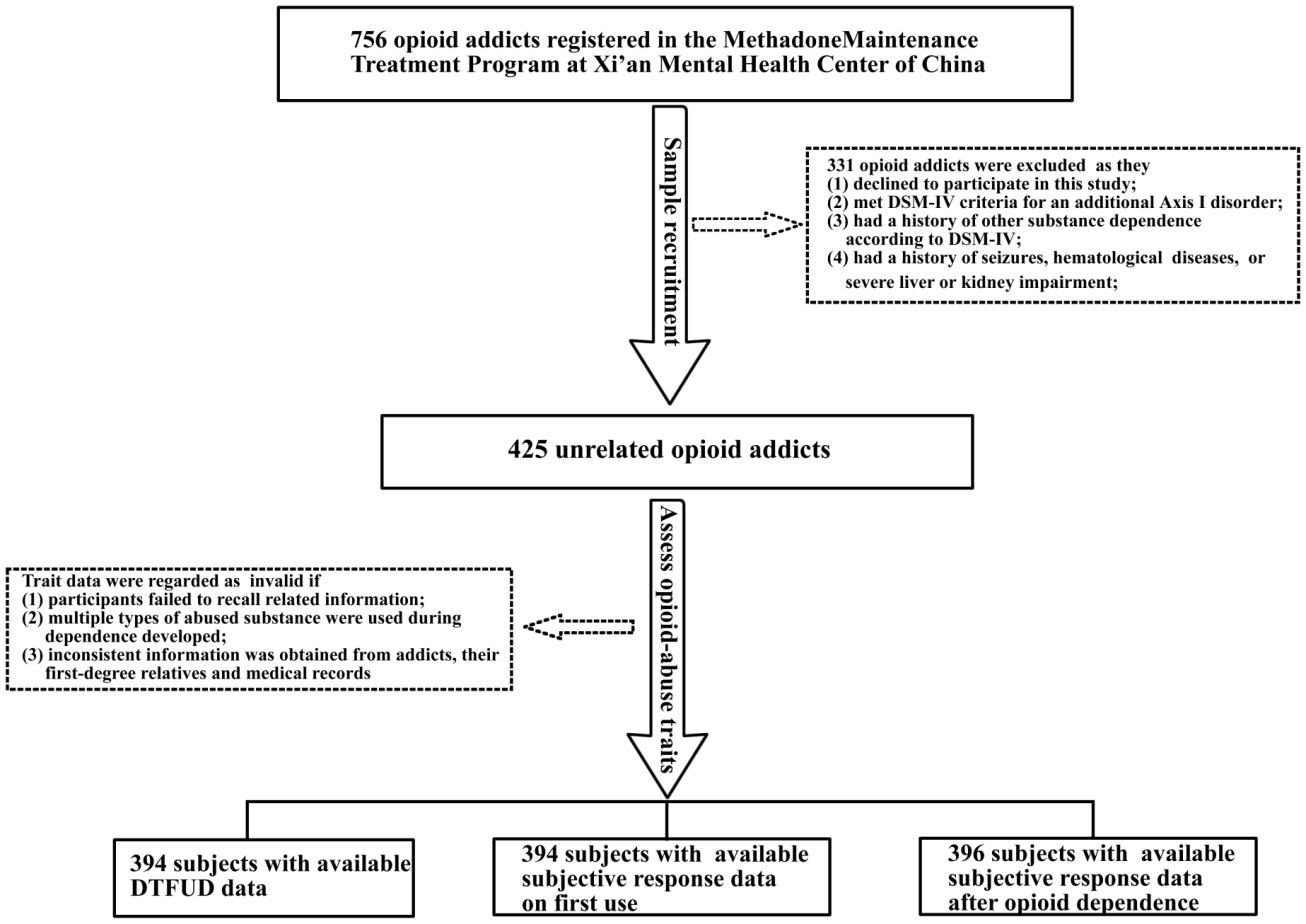
Opioid addicts recruitment and assessment of opioid-abuse traits.

The present study included 425 unrelated opioid addicts registered in the Methadone Maintenance Treatment Program at Xi'an Mental Health Center of China. The OD diagnosis was established using DSM-IV criteria and based on medical record, urine test, and interview (Fig. 1). The controls consisted of 514 unrelated healthy persons who had never been diagnosed with substance abuse and mental illness. All subjects participated voluntarily and signed written informed consent prior to the enrollment. This study was approved by the Medical Ethics Committee of Xi'an Jiaotong University.

### 2.2 Assessment of opioid-abuse traits

The following measures were obtained from medical records and a semi-structured interview: age at first opioid use, age of onset (AOO) of dependence, DTFUD, reasons for first opioid use, types of OD (heroin vs. opium poppy), administration route of first use and post-dependence use (nasal inhalation, intravenous or muscle injection), and opioid dosage. The DTFUD was defined as the duration from the initial opioid use to first occurrence of the dependence syndrome according to DSM-IV. The AOO and DTFUD were assessed by an interviewer and blindly verified by an independent psychiatrist using medical records and information provided by first-degree relatives, only the consistent data from these three sources were included in analysis (Fig. 1). Subjects who used drugs less than three times per week in the first month or could not obtain drugs for more than one week were excluded from the DTFUD analysis.

Subjective responses to opioid on first use and after dependence were examined using a method previously described with slight modification [13]. In brief, we asked the addicts to report their feelings on first-time opioid use and after the dependence using a checklist consisting of the following 17 items: “flushing,” “stimulated,” “numb,” “drunken,” “difficult to concentrate,” “drowsy,” “coasting or spaced out,” “turning of stomach,” “itchy skin,” “dry mouth,” “dizzy,” “nauseated,” “really high,” “carefree or happy,” “relaxed,” “euphoric,” and “without feeling effects of drug.” We classified the addicts into two groups on the basis of the feelings on first opioid use: comfortable (e.g., getting really high, happy, euphoric, and relaxed) vs. non-comfortable (e.g., turning of stomach, dizzy, nauseated, or no impact). We judged post-dependence responses to opioid based on subjective feelings occurring most frequently during use. The majority of addicts reported comfortable feelings, and only a small fraction of them declared that they could not feel any impact after developing OD. Based on the strength and frequency of pleasure, we assigned the addicts often feeling strong pleasure to the “euphoria” group and the addicts with low comfortable or no obvious feelings to the “non-euphoria” group.

### 2.3 Selection of polymorphisms

We first sequenced *DRD1* gene in 20 subjects randomly selected from the opioid addicts and screened out 8 SNPs with minor allele frequencies greater than 0.05. Our previous study [23] indicated perfect linkage disequilibrium (LD, r^2^ = 1) for two of the eight pairs of SNPs (rs265981 and rs686; rs10078714, and rs10078866). We therefore analyzed the remaining six SNPs in this study. We also included a well-characterized SNP in the Chinese population at the 3′ terminal of *DRD1* (rs4867798; HapMap-HCB database) for association analysis. The *DRD1* gene structure and the relative position of the seven SNPs are shown in Fig. S1.

### 2.4 Genotyping

We extracted genomic DNA from peripheral blood mononuclear cell using a TIANamp kit (Tiangen Biotech, Beijing, China). We amplified re-sequenced fragments by polymerase chain reaction and sequenced the products using an ABI 3730 DNA analyzer (Applied Biosystems, Foster City, CA, USA). We genotyped SNPs by MALDI-TOF MS using the MassARRAY system (Sequenom Inc., San Diego, CA, USA) [26]. Primers used for above experiments are shown in our previous study [23].

### 2.5 Statistical analysis

We calculated the power of the study to detect association with OD risk as previously described [27]. We analyzed categorical variables, such as gender, education level, OD type, route of drug administration, allele, genotype, and haplotype with the chi-square test. We used binary logistic regression to calculate the odds ratio (OR) and 95% confidence interval (CI) for each independent association, and to construct a model to predict the subjective response from polymorphisms and related covariates using a stepwise strategy.

We examined potential correlation of AOO and DTFUD with genotype and allele using Kaplan-Meier survival analysis. Survival curves estimated the probability that individuals had not experienced dependence over the period of time following first opioid use or at a certain age after birth. The DTFUD was regarded as survival time with the scale in days. The origin first is specified as the time of first opioid use, and the outcome of interest is first occurrence of the dependence syndrome. Since DTFUD in most addicts (>95%) was less than 360 days (see results below) and, the error of recalling increases with the self-reported DTFUD, we set the follow up time in survival analysis to a maximum of 360 days. Survival time is 360^+^ days when it exceeded 360 days. We compared survival curves using three methods (Wilcoxon, log rank, and Tarone–Ware tests) that give varying weight to different phases of follow-up time. We used a Cox proportional hazard regression model to test significant findings obtained with Kaplan–Meier analysis and to generate hazard ratios (HRs) and 95% CIs in a multivariate analysis, controlling for demographic and clinical features.

Hierarchical clustering on phenotype variables was used to identify subtypes, and for each subtype, logistic regression on subtype-control data was conducted to identify significant associations. We corrected multiple testing using a Bonferroni method: the *p*-value was divided by the total number of loci and considered significant at 0.0071. We performed intergroup comparison of genotype frequency based on codominant, heterosis, dominant, or recessive minor allele models of inheritance. We computed pair-wise LD statistics (D′ and r^2^) and haplotype frequency using Haploview 4.0 in order to construct haplotype blocks and to evaluate deviation from Hardy–Weinberg equilibrium (HWE) [28]. We used SPSS 16.0 software (SPSS Inc., Chicago, IL, USA) for statistical analyses.

## Results

### 3.1 Sample characteristics

Cases and controls were matched on age, gender, level of education, life with family, and unemployment (*p*>0.05, Table 1). Among the 425 opioid addicts, 133 initially developed dependence for opium poppy (Table 1). The DTFUD was available in 394 addicts, and ranged from 5 days to 11 years. Dependence was developed in 379 subjects within 360 days of first opioid use. We determined the subjective responses to opioid on first and post-dependence use in 394 and 396 addicts, respectively. A total of 64.0% addicts reported non-comfortable response upon first opioid use. The proportion of non-comfortable declined to 13.4% after the development of OD (*p* = 8.4×10^−35^), and all claimed no impact other than uncomfortable feelings. A total of 343 addicts reported pleasure response to opioid after dependence. We assigned 210 reporting frequent high pleasure after opioid use to the “euphoria” group. We assigned the remaining 133 low comfortable response addicts and the 53 no impact addicts to the “non-euphoria” group (Table 1).

**Table 1:** Demographic features of the controls and the opioid addicts stratified by the subjective responses on first use and post-dependence use.

Variable	Total cases	Response on first use (n = 394)		Response after OD (n = 396)	
		Comfortable	Non-comfortable	Euphoria	Non-euphoria
Number	425	138(35.0)	256(65.0)	210(53.0)	186(47.0)
Male gender (%)	365 (85.9)	126 (91.3)	217 (84.8)	179 (85.2)	166 (89.2)
Education					
≤Junior high school (%)	267 (63.1)	88 (63.8)	151 (59.0)	127 (60.5)	113 (60.8)
≥Senior high school (%)	156 (36.9)	50 (36.2)	105 (41.0)	83 (39.5)	73 (39.2)
Live with family (%)	295 (69.4)	92 (66.7)	176 (68.8)	143 (68.1)	126 (67.7)
The unemployed (%)	269 (63.3)	91(65.9)	149 (58.2)	131 (62.4)	111 (59.7)
Age of onset (mean±SD)^a^	26.5±6.6	26.8±6.4	26.4±6.6	26.6±6.5	26.4±6.6
Reason for first use of opioid					
Curiousness (%)	222 (55.8)	85 (61.6)	135 (52.7)	110 (52.4)	112 (60.2)
Peer pressure (%)	68 (17.1)	**11 (8.0)** b	**56 (21.9)**	36 (17.2)	31 (16.7)
Trouble (%)	64 (16.1)	24 (17.4)	39 (15.2)	38 (18.1)	25 (13.4)
Entertainment (%)	24 (6.0)	10 (7.2)	14 (5.5)	15 (7.1)	9 (4.8)
Physical disease (%)	8 (2.0)	3 (2.2)	5 (2.0)	4 (1.9)	4 (2.2)
Others reasons (%)	12 (3.0)	5 (3.6)	7 (2.7)	7 (3.3)	5 (2.7)
Type of OD					
Heroin (%)	292 (68.7)	86 (62.3)	175 (69.2)	137 (66.2)	124 (67.0)
Opium poppy (%)	133 (31.3)	52 (37.4)	78 (30.8)	70 (33.8)	61 (33.0)
Method of first opioid use					
Sniffed or smoked (%)	374 (88.0)	**122 (88.4)**	**248 (96.9)**	200 (95.2)	172 (92.5)
Injection via vein (%)	21 (4.9)	**13 (9.4)**	**8 (3.1)**	7 (3.3)	9 (4.8)
Ineligibles (%)	30 (7.1)	3 (2.2)	0 (0.0)	3 (1.5)	5 (2.7)
Method of opioid use after OD					
Sniffed or smoked (%)	127 (32.5)	33 (23.9)	93 (36.5)	63 (30.1)	64 (34.4)
Injection via vein (%)	194 (49.6)	80 (58.0)	111 (43.5)	103 (49.3)	89 (47.9)
Others (%)	70 (17.9)	25 (18.1)	51 (20.0)	43 (20.6)	33 (17.7)
DTFUD (day, median (IQR))	30 (60)	60 (90)	30 (106)	60 (90)	30 (99)

a Age means the age at the onset of opioid dependence for the cases and the age when they participated in our study for the controls.

b The values of the variable with significant *p*-value are marked in bold.

### 3.2 Association of *DRD1* polymorphism with AOO and DTFUD

The *DRD1* polymorphism was not associated with collective AOO of OD (combined heroin and opium poppy dependence, *p*>0.05). The AOO was significantly earlier for opium poppy dependence (n = 130, median AOO: 23.4 years) than for heroin dependence (HD, n = 261, median AOO: 28.1 years, *p*<8.3×10^−13^ for log rank, Tarone–Ware and Wilcoxon tests). A Kaplan–Meier survival analysis indicated that only the rs10063995 genotype was associated with AOO for HD (*p*<0.003 for log rank, Tarone–Ware, and Wilcoxon tests). Heroin-dependent subjects carrying the TT genotype (n = 23, median AOO: 24.2 years) had earlier AOO than those carrying GG or GT genotype (n = 240, median AOO: 28.5 years).

**Figure 2:**
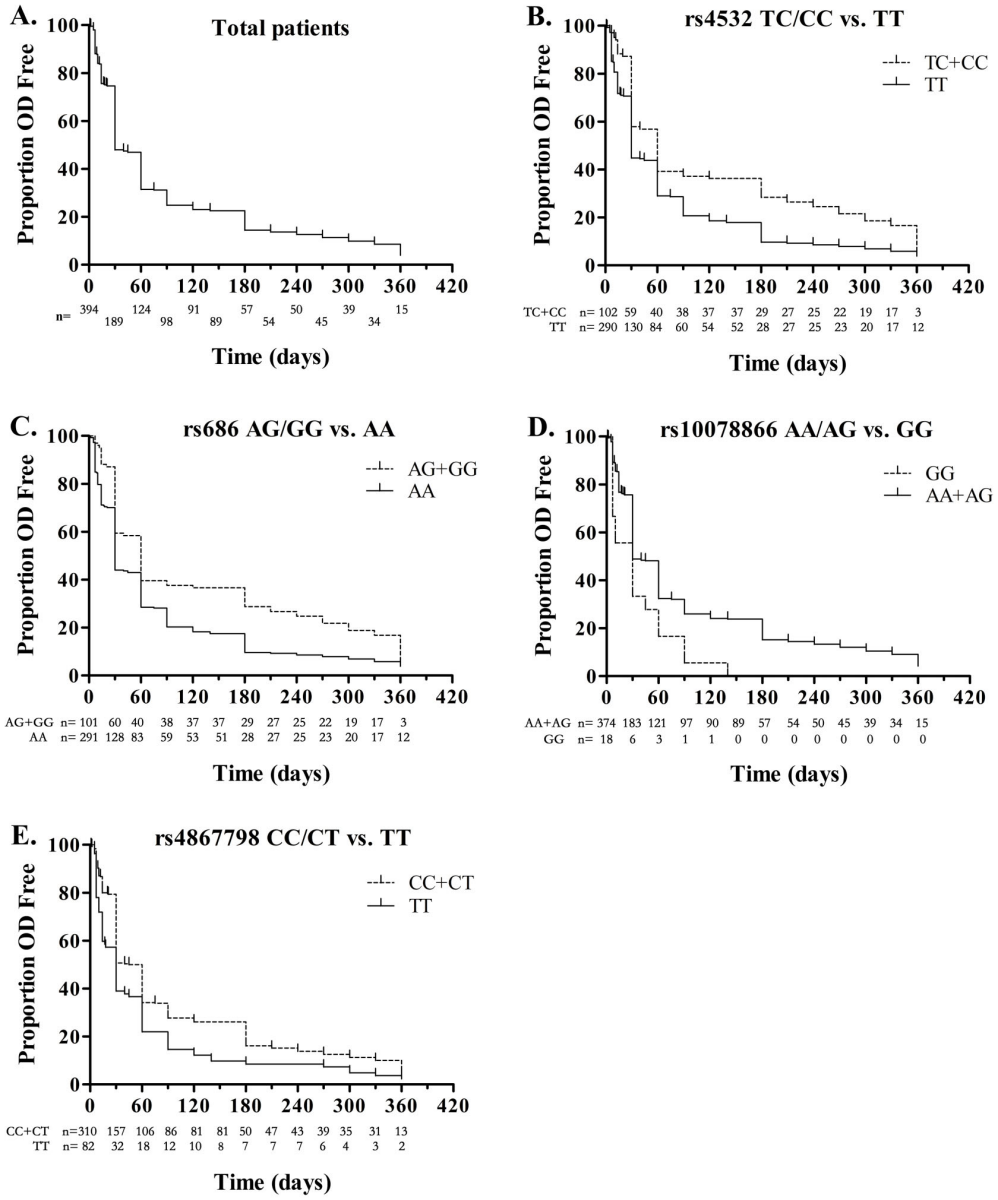
Kaplan–Meier survival curves representing probability that individuals have not experienced dependence over the period of time following first opioid use. Total addicts (A), stratified by combined minor allele homozygote and heterozygote vs. major allele of rs4532 homozygote (B), by combined minor allele homozygote and heterozygote vs. major allele of rs686 homozygote (C), by combined major allele homozygote and heterozygote vs. minor allele of rs10078866 homozygote (D), by combined major allele homozygote and heterozygote vs. minor allele of rs4867798 homozygote (E). The numbers of patients who have not experienced dependence at a certain time are shown below x-axis for each genotype.

The probability for emergence of dependence at a certain time after first opioid use is shown in Fig. 2A. We used Kaplan–Meier survival analysis to detect the potential influences of *DRD1* polymorphisms and demographic and clinical features on the DTFUD (Table 2). Subjects carrying the minor allele of rs4532 (Fig. 2B) or rs686 (Fig. 2C) had prolonged DTFUD in comparison to homozygotes (*p*<0.001 for each SNP using log rank, Tarone–Ware, and Wilcoxon tests). We used AOO, OD type, response after first opioid use, gender, as covariates for a Cox regression. The minor allele-carrying genotypes of rs4532 and rs686 were significantly associated with lower risk to develop dependence after first opioid use compared to homozygotes (rs4532 HR = 0.694; rs686 HR = 0.681). Shorter DTFUD was noted in minor allele homozygotes with rs10078866 (Fig. 2D) and rs4867798 (Fig. 2E; *p*<0.005 for each SNP using log rank, Tarone–Ware, and Wilcoxon tests). There was a significant increased risk to develop dependence after first opioid use with GG genotype of rs10078866 (vs. AA+AG genotype, HR = 1.875) and TT genotype of rs4867798 (vs. CC+CT genotype, HR = 1.478). The association between rs4532 and rs686 and DTFUD was also evident in a more homogeneous group of 262 HD subjects (results not shown).

**Table 2:** Associations of DTFUD with *DRD1* polymorphisms and demographic and clinical features.

Variable	n	DTFUD		*p*-value^a^			Adjusted^b^ HR (95% CI)
		Median	IQR	LR	TW	Wil	
rs10078866				0.008	0.014	0.021	
AA	247	30	150	0.118	0.121	0.143	
AG	127	60	76	0.131	0.155	0.185	
GG	18	30	53	**0.003**	**0.005**	0.008	**1.875 (1.163∼3.023)**
rs10063995				0.226	0.256	0.275	
GG	254	30	150	0.263	0.322	0.429	
GT	116	45	69	0.051	0.035	0.026	
TT	23	30	81	0.110	0.117	0.115	
rs5326				0.217	0.203	0.223	
GG	228	30	90	0.372	0.417	0.484	
GA	140	30	69	0.476	0.386	0.386	
AA	24	30	53	0.092	0.079	0.086	
rs4532				**0.002**	**0.001**	**0.001**	
TT	290	30	76	**0.001**	**0.0002**	**0.0002**	**0.694 (0.548∼0.878)**
TC	89	60	180	0.192	0.118	0.100	
CC	13	60	270	0.094	0.060	0.060	
rs1799914							
GG	333	30	60	0.816	0.743	0.662	
GA+AA^c^	59	30	170				
rs686				**0.001**	**0.0003**	**0.0004**	
AA	291	30	76	**0.0003**	**0.00009**	**0.00008**	**0.681 (0.538∼0.862)**
AG	88	60	180	0.237	0.165	0.150	
GG	13	60	270	0.092	0.058	0.057	
rs4867798				**0.001**	**0.001**	**0.001**	
CC	113	30	60	0.765	0.934	0.733	
CT	197	60	160	0.804	0.965	0.931	
TT	82	30	50	**0.0005**	**0.0003**	**0.0002**	**1.478 (1.151∼1.896)**
Types^d^							
Heroin	258	30	166	0.070	0.261	0.561	
Opium	132	30	72				
Response^d^							
Negative	252	30	76	0.241	0.243	0.233	
Positive	138	60	90				
Gender							
Male	343	30	72	0.794	0.923	0.793	
Female	51	60	110				

a For genetic variants, *p*-value was calculated based on codominant, dominant for minor allele, heterosis and recessive for minor allele models of inheritance, respectively. LR: Log Rank test; TW: Tarone–Ware test; Wil.: Wilcoxon test.

b Only the positive factors found by Kaplan–Meier were included into Cox regression models to calculate HRs, which were adjusted for AOO, gender, types of initial OD, and subjective response on first opiate use.

c Since there were a small amount of subjects carrying the AA genotype of rs1799914, the AA and GA genotypes were merged to conduct the statistical test.

d Types: types of OD; Response: subjective response on first opiate use.

### 3.3 Polymorphisms and the subjective responses to opioid

Most demographic and opioid abuse-related characteristics were comparable between different response categories, except for the reason and opioid route of first use (Table 1). The proportion of peer pressure as the reason for the opioid use initiation was lower in subjects with comfortable response than in those with non-comfortable response (8% vs. 21.9% respectively, *p* = 0.0005). With respect to administration route of first drug use, the frequency of intravenous injection in subjects with comfortable response was larger than that in non-comfortable response subjects (9.4% vs. 3.1%, *p* = 0.007).

We compared the genotype and allele frequencies of *DRD1* polymorphisms between groups with different responses to opioid (Table 3). Allele and/or genotype frequencies at rs5326, rs10063995, and rs10078866 differed significantly between those with comfortable vs. non-comfortable responses on first opioid use (*p*<0.0071). Decreased risk to develop comfortable response to opioid after first use was observed in subjects carrying the GA+AA genotype (OR = 0.500) and A allele (OR = 0.551) of rs5326, the GT+TT genotype (OR = 0.373), GT genotype (OR = 0.318), and T allele (OR = 0.502) of rs10063995, as well as the AG+GG genotype (OR = 0.516) and AG genotype (OR = 0.488) of rs10078866. The above associations remained significant after adjustment for AOO, gender, first opioid use reason, initial OD type, and first opioid use methods. Moreover, the opioid use route (intravenous injection vs. sniffing, OR = 3.605, 95% CI: 1.391∼9.346) is a predictive factor for opioid-induced comfortable responses.

**Table 3:** Association between *DRD1* polymorphisms and the subjective responses to opioid.

		Response on	first opioid	use		Response	after	dependence
Variable	Comfortable(n = 138)	Non-comfortable(n = 256)	χ^2^ analysis *p*-value^a^	Logistic regression^b^ OR, 95%CI	Euphoria (n = 210)	Non-euphoria(n = 186)	χ^2^ analysis *p*-value^a^	Logistic regression^b^ OR, 95%CI
rs10078866			0.023				0.025	
AA	99(71.7)	147(57.9)	**0.007**	**0.516, 0.323∼0.824**	118(56.7)	130(69.9)	**0.007**	1.758, 1.127∼2.743
AG	33(23.9)	94(37)	0.008	**0.488, 0.299∼0.798**	78(37.5)	49(26.3)	0.018	1.635, 1.045∼2.560
GG	6(4.3)	13(5.1)	0.734	1.020, 0.350∼2.972	12(5.8)	7(3.8)	0.354	1.122, 0.408∼3.081
Per G allele	45(16.3)	120(23.6)	0.016	0.621, 0.417∼0.927	102(24.5)	63(16.9)	0.009	1.494, 1.031∼2.164
rs10063995			0.0004				0.433	
GG	106(76.8)	147(57.6)	**0.0002**	**0.373, 0.228∼0.611**	129(61.7)	125(67.2)	0.256	1.274, 0.817∼1.988
GT	24(17.4)	92(36.1)	**0.0001**	**0.318, 0.185∼0.548**	65(31.1)	52(28)	0.495	1.197, 0.759∼1.886
TT	8(5.8)	16(6.3)	0.850	0.938, 0.363∼2.422	15(7.1)	9(4.8)	0.332	1.108, 0.447∼2.750
Per T allele	40(14.5)	124(24.3)	**0.001**	**0.502, 0.331∼0.759**	95(22.7)	70(18.8)	0.177	1.202, 0.832∼1.737
rs5326			0.013				0.440	
GG	92(67.2)	136(53.3)	0.008	**0.500, 0.317∼0.789**	117(56)	112(60.5)	0.360	1.211, 0.791∼1.854
GA	41(29.9)	98(38.4)	0.093	0.572,0.356∼0.917	76(36.3)	64(34.6)	0.714	1.106, 0.717∼1.706
AA	4(2.9)	21(8.2)	0.040	0.410, 0.132∼1.273	16(7.7)	9(4.9)	0.257	1.227, 0.502∼2.997
Per A allele	49(19.9)	140(27.5)	**0.003**	**0.551, 0.375∼0.809**	108(25.8)	82(22.2)	0.229	1.170, 0.828∼1.653
rs4532			0.948					
TT	102(74.5)	188(73.7)	0.876	0.822, 0.498∼1.356	169(80.9)	122(65.9)	**0.0008**	**0.431, 0.266∼0.698**
TC	31(22.6)	58(22.7)	0.978	0.814, 0.481∼1.379	33(15.8)	57(30.8)	**0.0004**	**0.391, 0.235∼0.649**
CC	4(2.9)	9(3.5)	0.748	0.942, 0.280∼3.166	7(3.3)	6(3.2)	0.953	1.078, 0.330∼3.519
Per C allele	39(14.2)	76(14.9)	0.801	0.857, 0.556∼1.322	47(11.2)	69(18.6)	**0.003**	**0.537, 0.355∼0.812**
rs1799914								
GG	114(83.2)	221(86.7)	0.355	1.324, 0.722∼2.430	174(83.3)	162(87.6)	0.228	1.337, 0.735∼2.432
GA+AA	23(16.8)	34(13.3)			35(16.7)	23(12.4)		
Per A allele	24(8.7)	34(6.7)	0.286	1.356, 0.765∼2.401	35(8.4)	24(6.5)	0.315	1.254, 0.713∼2.205
rs686			0.920				**0.001**	
AA	102(73.9)	189(74.4)	0.915	0.889, 0.541∼1.461	169(81.3)	123(66.1)	**0.0006**	**0.425,0.262∼0.689**
AG	32(23.2)	56(22)	0.796	0.891, 0.528∼1.504	32(15.4)	57(30.6)	**0.0003**	**0.385, 0.231∼0.641**
GG	4(2.9)	9(3.5)	0.733	0.926, 0.275∼3.111	7(3.4)	6(3.2)	0.938	1.101, 0.338∼3.591
Per G allele	40(14.5)	74(14.6)	0.978	0.907, 0.589∼1.396	46(11.1)	69(18.5)	**0.003**	**0.535, 0.353∼0.810**
rs4867798			0.036				0.973	
TT	37(27)	78(30.6)	0.458	1.156, 0.704∼1.897	62(29.7)	53(28.6)	0.825	0.991, 0.621∼1.582
CT	79(57.7)	115(45.1)	0.018	1.595, 1.018∼2.501	103(49.3)	93(49.3)	0.845	0.992, 0.647∼1.521
CC	21(15.3)	62(24.3)	0.038	0.593, 0.336∼1.046	44(21.1)	39(21.1)	0.994	0.946, 0.563∼1.591
Per T allele	121(44.2)	239(46.9)	0.469	0.898, 0.660∼1.221	191(45.7)	171(46.2)	0.883	0.979, 0.729∼1.314

a *p*-value was calculated by 2×3 and 2×2 chi-squared tests (for codominant, dominant for rare allele, heterosis, recessive for rare allele, and allele model). *p*-values were adjusted by Bonferroni correction and statistically significant results (*p*<0.0071) are marked in bold.

b Demographic variables (gender, AOO, types of opiate dependence, methods of first opiate use) are controlled. The ORs with statistically significant results (*p*<0.071) after Bonferroni correction are marked in bold.

For the response after dependence, the allele and genotype frequency of rs4532 and rs686 was significantly different between euphoria and non-euphoria groups (Table 3, *p*<0.003). The decreased risk to acquire opioid-induced euphoria after dependence was associated with the AG+GG genotype (OR = 0.425), AG genotype (OR = 0.385) and G allele (OR = 0.535) of rs686, the TC+CC genotype (OR = 0.431), TC genotype (OR = 0.391) and C allele (OR = 0.537) of rs4532, after adjustment for AOO, gender, and opioid use route.

### 3.4 Polymorphisms and the OD risk

No significant HWE deviation was found for any SNP in cases and controls (*p*>0.05). The pair-wise LD values of these SNPs and their haplotype structure were similar to our previous published results (data not shown) [23]. An analysis of the genotype and allele frequencies suggested only a trend-level association between rs686 and OD (*p* = 0.008∼0.025 for different inheritance model; *p*>0.0071 after Bonferroni correction) (Table 4). We estimated the power to detect association with OD in this sample size (425 cases and 514 controls) to be 76–99%, assuming an effect size of 1.6 at a nominal *p* = 0.05 for minor allele frequencies ranging from 0.06 to 0.44 (65). An analysis stratified by efficiency of transition to dependence and subjective response, gender, AOO, and type of initial OD revealed a significant association between *DRD1* and OD with fast transition (DTFUD≤30 days), OD with first comfortable response, as well as OD with post-dependence euphoria (Table 4). The subjects carrying the AG, AG+GG genotype, or G allele of rs686 had decreased risk for OD with fast transition (OR _GA_ = 0.524, 95% CI: 0.352∼0.782; OR _GA+AA_ = 0.537, 95% CI: 0.365∼0.790; OR _A allele_ = 0.603, 95% CI: 0.426∼0.853) and OD with post-dependence euphoria (OR _GA_ = 0.427, 95% CI: 0.280∼0.650; OR _GA+AA_ = 0.490, 95% CI: 0.330∼0.727; OR _A allele_ = 0.603, 95% CI: 95% CI: 0.426∼0.853) in comparison to the subjects carrying GG+AA, GG genotypes or G allele, respectively. The CT and CT+TT genotypes of rs10063995 were associated with decreased risk of developing OD with first comfortable response (OR _CT_ = 0.472, 95% CI: 0.292∼0.761; OR _CT+TT_ = 0.525, 95% CI: 0.340∼0.810) in comparison to the CC+TT and CC genotypes, respectively. The TC and TC+CC genotype of rs4532 were associated with OD with post-dependence euphoria (OR _TC_ = 0.486, 95% CI: 0.320∼0.740; OR _TC+CC_ = 0.553, 95% CI: 0.374∼0.820) in comparison to the AA+GG and AA genotypes, respectively.

**Table 4:** Association between *DRD1* polymorphisms and the risk of OD.

Variable	Controls (n = 514)	Cases (n = 425)	*p*-value^a^	Fast transition (n = 209)	*p*-value	First comfortable (n = 138)	*p*-value	Euphoria after OD (n = 210)	*p*-value
rs10078866			0.942		0.378		0.096		0.308
AA	320(62.4)	265(62.6)	0.932	131(63.6)	0.820	99(71.7)	0.042	118(56.7)	0.159
AG	172(33.5)	139(32.9)	0.829	63(30.4)	0.423	33(23.9)	0.031	78(37.5)	0.310
GG	21(4.1)	19(4.5)	0.764	13(6.3)	0.211	6(4.3)	0.894	12(5.8)	0.329
Per G allele	214(20.9)	177(20.9)	0.973	89(21.5)	0.787	45(16.3)	0.093	102(24.5)	0.128
rs10063995			0.929		0.670		**0.0069**		0.729
GG	325(63.5)	274(64.6)	0.716	136(65.4)	0.629	106(76.8)	**0.003**	129(61.7)	0.658
GT	158(30.9)	126(29.7)	0.705	58(27.9)	0.430	24(17.4)	**0.002**	65(31.1)	0.949
TT	29(5.7)	24(5.7)	0.998	14(6.7)	0.584	8(5.8)	0.952	15(7.2)	0.441
Per T allele	216(21.1)	174(20.5)	0.760	86(20.7)	0.859	40(14.5)	0.014	95(22.7)	0.494
rs5326			0.717		0.897		0.173		0.563
GG	304(59.8)	242(57.2)	0.417	120(58)	0.644	92(67.2)	0.119	117(56)	0.340
GA	173(34.1)	154(36.4)	0.454	74(35.7)	0.666	41(29.9)	0.362	76(36.4)	0.555
AA	31(6.1)	27(6.4)	0.860	13(6.3)	0.928	4(2.9)	0.144	16(7.7)	0.445
Per A allele	235(23.1)	208(24.6)	0.462	100(24.2)	0.678	49(17.9)	0.063	108(25.8)	0.275
rs4532			0.132		0.056		0.433		**0.002**
TT	360(70)	315(74.5)	0.133	163(78.7)	0.018	102(74.5)	0.312	169(80.9)	**0.003**
TC	143(27.8)	95(22.5)	0.061	40(19.3)	0.018	31(22.6)	0.222	33(15.8)	**0.001**
CC	11(2.1)	13(3.1)	0.368	4(1.9)	0.860	4(2.9)	0.589	7(3.3)	0.344
Per C allele	165(16.1)	121(14.3)	0.295	48(11.6)	0.031	39(14.2)	0.462	47(11.2)	0.019
rs1799914									
GG	444(87.4)	359(84.9)	0.264	176(85)	0.396	114(83.2)	0.203	174(83.3)	0.143
AA+ GA^b^	64(12.6)	64(15.1)		31(15)		23(16.8)		35(16.7)	
Per A allele	65(6.4)	65(7.7)	0.278	32(7.7)	0.364	24(8.8)	0.171	35(8.4)	0.182
rs686			0.025		**0.005**		0.282		**0.0002**
AA	348(68)	316(74.7)	0.024	166(79.8)	**0.001**	102(73.9)	0.179	169(81.3)	**0.0003**
AG	153(29.9)	94(22.2)	0.008	38(18.3)	**0.001**	32(23.2)	0.122	32(15.4)	**0.0001**
GG	11(2.1)	13(3.1)	0.373	4(1.9)	0.848	4(2.9)	0.602	7(3.4)	0.343
Per G allele	175(17.1)	120(14.2)	0.086	46(11.1)	**0.004**	40(14.5)	0.303	46(11.1)	**0.004**
rs4867798			0.796		0.264		0.271		
TT	153(30.1)	120(28.4)	0.559	60(29)	0.764	37(27)	0.478	62(29.7)	0.917
TC	255(50.2)	214(50.6)	0.905	95(45.9)	0.297	79(57.7)	0.121	103(49.3)	0.904
CC	100(19.7)	89(21)	0.609	52(25.1)	0.107	21(15.3)	0.246	44(21.1)	0.824
Per T allele	455(44.8)	392(46.3)	0.503	199(48.1)	0.258	121(44.2)	0.854	191(45.7)	0.678

a *p*-value was calculated by 2×3 and 2×2 chi-squared tests based on codominant, dominant for minor allele, heterosis, and recessive for minor allele models of inheritance, respectively.

b Since there were a small amount of subjects carrying the AA genotype of rs1799914, the AA and GA genotypes were merged to conduct 2×2 chi-squared tests.

We also performed a multivariate cluster analysis on the 398 addicts using 16 clinical measures that characterized their opioid use and related behaviors, mainly including the AOO, gender, type of opioid, administration route, daily administration dosage of opioid before methadone treatment, use times of opioid daily before methadone treatment, DTFUD, subjective response, and so on. The cluster analysis resulted in four subgroups of addicts that were more homogeneous with respect to these clinical measures within each group (Fig. S2). In particular, these clusters differed significantly on the subjective responses to opioid, respectively, at the initial use and after the development of dependence on opioid. The percentages of subject experiencing comfortable response to opioid initially subgroup1-4 were 99%, 0, 0, 34%, respectively. And the percentages of subject experiencing euphoric response to opioid after dependence in subgroup1-4 were 59%, 98%, 1%, 39%, respectively. We then conducted association tests for each of the subgroups to identify subgroup-dependent genetic effects that may be overlooked when all addicts were considered in one group. Table 5 shows the test results for the detection of main effects. DRD1 rs686 and rs4532 displayed significant association with subtype 2 and/or subtype 4.

**Table 5:** Results of association analysis between *DRD1* SNPs and subtypes of OD.

Varible	rs10078866	rs10063995	rs5326	rs4532	rs1799914	rs686	rs4867798
Subtype1	0.316	0.145	0.139	0.021	0.161	0.011	0.860
Subtype2	0.018	0.064	0.024	0.027	0.267	**0.006** *	0.288
Subtype3	0.785	0.498	0.245	0.930	0.613	0.649	0.305
Subtype4	0.055	0.126	0.297	**0.003** *	0.816	**0.007** *	0.394

* indicates the significant results after Bonferroni multi-test correction.

## Discussion

### 4.1 Fast transition from first opioid use to dependence and opioid-induced pleasure response affects predisposition to OD

In the present study, no statistically significant association was found between *DRD1* and OD in the overall analysis. However, the association became apparent after stratifying based on DTFUD (faster transition) and opioid-induced subjective response (pleasure response on first use or post-dependence euphoria). This finding supported the previous observation that DSM-IV OD classification is not optimal for genetic mapping [3], [4], [5], and suggests that fast transition to dependence, pleasure responses upon first-time use, and post-dependence euphoria are more heritable traits.

Inheritable predisposition to drug dependence comprises vulnerability in both initiation of drug use and the transition from first use to abuse to dependence [2], [29]. Both animal experiments [8], [9] and epidemiological investigation [6] indicated that only a small part of subjects who have taken drugs for a prolonged period develop dependence. The proportion of the drug users who develop dependence within 1 year and 10 years after first use is 1% and 12–13% respectively for alcohol, 1.5% and 8% respectively for marijuana, and 5.5% and 15–16% respectively for cocaine [6]. Apparently, people with rapid progression from initial use to compulsive use are more likely to develop dependence [10]. Accordingly, genetic factors that place certain individuals at faster (or slower) transition more likely promote (or prevent) them to develop dependence.

Inter-individual variation in the reward process is associated with personality and temperament [30] as well as susceptibility to drug dependence [31]. People use drugs mostly due to their inherent rewarding property, particularly prior to the development of compulsive use [12]. In general, people with stronger pleasure responses to drugs have higher vulnerability to eventual dependence.

### 4.2. Role of *DRD1* polymorphisms on the transition from first opioid use to dependence

Animal experiments indicate drug dosage is an important determinant for addiction development [32]. The time length but not accumulative opioid intake amount was selected as the index for efficiency of transition, because it is the onset time of a specific event that could be more reliably and accurately recalled than dose in human retrospective study. To further elevate the reliability and validity of time assessment, we also executed cross-examination for self-reported information, including that from lineal relatives and medical records. Drug availability affects the measure of DTFUD for transition to dependence. Restriction of personal freedom and being poor increase DTFUD, but not due to decreased transition efficiency [2], [10]. The DTFUD analysis included only subjects who used opioid at a frequency of three or more times per week in the first month after the first use and could readily obtain the drug to fulfill high validity to measure the efficiency of transition to dependence.

We found a large variation of DTFUD from 5 days to 11 years. Our data indicated an association between minor alleles of rs4532 and rs686 and longer DTFUD. Moreover, the homozygote addicts with the rs10078866 and rs4867798 minor alleles have shorter DTFUD than non-carriers. These findings represent the first direct evidence of the influence of transition to dependence by genetic factors. Notably, rs686 is a functional polymorphism that influences the expression of *DRD1* [25]. The genetic variation of rs686 from A to G decreases *DRD1* expression, and has been associated with a variety of dopamine-related diseases [20], [23], [24].

Suppressing D1 receptor function could decrease the efficiency of transition to dependence. For example, D1 receptor genetic knockout or pharmacological blockade inhibits cocaine [33] and heroin self-administration [34]. Morphine self-administration is also decreased by dopaminergic antagonists [35]. In contrast, the D1 receptor agonist SKF82958 enhances heroin self-administration in rhesus monkeys [36].

Stimulation of D1 receptors by opioid initiates a sequence of molecular events, including c-Fos [37], ΔFosB [38], [39], ERK [40] and CREB [41] activation, which shape neuron structure and function [42]. Drug-induced persistent neuroadaptation in reward-related learning and memory processes, which leads to hypersensitivity to drug-associated cues, impulsive decision-making and abnormal learned behaviors, are the neurobiological basis for the transition to dependence [42], [43]. A postmortem analysis revealed the regulatory effects of *DRD1* variation on its mRNA expression in striatum, which were blunted by chronic opioid abuse [19]. In addition, there is an association between *DRD1* variation and HOMER1b/c protein in the human striatum; its pattern also varies between opioid abusers and healthy controls [19]. These findings demonstrate that an interaction between *DRD1* variation and opioid action exists to influence neuroplasticity. Presumably, the variation of rs686 from A to G prolongs DTFUD by suppressing the function of D1 receptor to interfere with the activation of downstream transcriptional factors and the long-term neuroplasticity in brain motivation and reward-related circuits induced by repeated opioid use. We propose that rs686 is more likely to be a causative SNP for the altered DTFUD rather than a marker. The positive association of rs4532 with DTFUD can be attributed to its complete linkage disequilibrium with rs686 (r^2^ = 0.96).

### 4.3 The effects of polymorphisms in *DRD1* on subjective response to opioid

Our data indicates that polymorphisms in *DRD1* modulate subjective responses to opioid in both the initial phase and post-dependence. Decreased pleasure responses on first use were associated with the minor allele-carrying genotypes rs5326, rs10063995, and rs10078866. Decreased euphoria after dependence was associated with the minor alleles of rs686 and rs4532, as well as the genotypes containing these minor alleles. A previous study [13] demonstrated that subjective responses induced by first-used heroin were affected by the genetic variants in μ-opioid receptor gene. After μ-opioid receptor is activated by opioid, the dopamine neurons in ventral tegmental area (VTA) are dis-inhibited via inhibiting GABAergic interneurons in VTA to induce synaptic dopamine release and the activation of dopamine receptor in the brain reward circuit [44]. Genetic variation in dopamine neurotransmission is assumed to alter central dopaminergic tone and the reward process [18]. Our results provide strong evidence that the opioid reward process can be influenced by *DRD1* genetic variants.

Consistent with a previous report [13], we found that the majority of addicts did not have apparent pleasant feeling upon first opioid use. In contrast, most addicts felt euphoria upon exposure after the development of dependence, suggesting enhanced sensitivity to opioid rewarding effects. Animal experiments confirmed reward sensitization induced by repeated exposure occurs for a variety of drugs of abuse, which is mediated by several molecules upregulation in amygdala and striatum [45], [46], [47]. The enhanced pleasure responses to opioid after dependence in humans are similar to reward sensitization in animal, which, depending on the chronic drug-induced neuroplastic changes, specifically upregulates reward function. Moreover, different opioid reward encoding patterns were identified between drug-naïve and dependent rats and the role of D1 receptor transmission in reward also depends on opioid state [48]. The roles of DRD1 in pleasure process of first opioid use and post-dependence use may be different, which also explains why initial pleasure-associated variants are different from post-dependence pleasure-associated variants. DRD1-dependent signaling mediates many long-term neuroadaptation in reward circuit which may participate in opioid reward after dependence [38], [39], [41], [48]. Down-regulating DRD1 using a viral-mediated siRNA in the nucleus accumbens decreases ethanol-induced behavioral sensitization as well as ethanol rewarding properties [49]. Since the G allele of rs686 decreases the expression of *DRD1*, we speculate that the signaling of D1 receptor upon repeated opioid exposure is blunted by the G allele of rs686, so the G allele carrier displayed significantly decreased reward compared with the A allele.

### 4.4 *DRD1* polymorphisms and OD risk

Jacobs et al. [19] recently reported a trend-level association between rs686 and OD in Caucasians (*p* = 0.02). Our comparison between opioid addicts and controls also revealed a possible association between rs686 and OD risk (*p* = 0.008∼0.025 for different inheritance model), which was not significant after Bonferroni correction. Instead, we carried out a probabilistic method using different strategy from chi-square test to detect potential association [50], and still revealed no significant effects of *DRD1* on OD risk (*p*>0.05, data not shown). The *DRD1* rs686 affected the disease susceptibility only in a subgroup of addicts with fast transition to dependence or euphoria, suggesting the heritable predisposition to OD is dependent on the varying subtype and features of dependence. The OD subtype with fast transition to dependence or euphoria had a greater genetic load than overall OD. The effect size of rs686 specific for these OD subtypes was decreased by heterogeneous subtype, which correspondingly decreased power of genetic association analysis. In conclusion, *DRD1* rs686 minor allele decreases OD risk by slowing down the transition to dependence and attenuating opioid-induced euphoria.

## Supporting Information

Figure S1 **Gene structure of human** ***DRD1*** **, showing the re-sequencing fragments and the relative positions of the 7 SNPs used in our study.** The black squares above the chart of gene structure indicate the fragments we targeted for re-sequencing.
(TIF)

Figure S2 **Four subgroups of addicts defined by a multivariate cluster analysis.** The columns with different color indicate the different clinical measures that characterized opioid use and related behaviors, mainly including the age of onset, gender, type of opioid, administration route, daily administration dosage of opioid before methadone treatment, use times of opioid daily before methadone treatment, DTFUD, subjective response, and so on.
(TIF)

## References

[1] Tsuang MT, Lyons MJ, Eisen SA, Goldberg J, True W, et al (1996) Genetic influences on DSM-III-R drug abuse and dependence: a study of 3,372 twin pairs. Am J Med Genet 67: 473–477. 8886164 10.1002/(SICI)1096-8628(19960920)67:5<473::AID-AJMG6>3.0.CO;2-L

[2] Bierut LJ (2011) Genetic vulnerability and susceptibility to substance dependence. Neuron 69: 618–627. 21338875 10.1016/j.neuron.2011.02.015 PMC3095110

[3] Gelernter J, Panhuysen C, Wilcox M, Hesselbrock V, Rounsaville B, et al (2006) Genomewide linkage scan for opioid dependence and related traits. Am J Hum Genet 78: 759–769. 16642432 10.1086/503631 PMC1474044

[4] Chan G, Gelernter J, Oslin D, Farrer L, Kranzler HR (2011) Empirically derived subtypes of opioid use and related behaviors. Addiction 106: 1146–1154. 21306596 10.1111/j.1360-0443.2011.03390.x PMC3164489

[5] Sun J, Bi J, Chan G, Oslin D, Farrer L, et al (2012) Improved methods to identify stable, highly heritable subtypes of opioid use and related behaviors. Addict Behav 37: 1138–1144. 22694982 10.1016/j.addbeh.2012.05.010 PMC3395719

[6] Wagner FA, Anthony JC (2002) From first drug use to drug dependence; developmental periods of risk for dependence upon marijuana, cocaine, and alcohol. Neuropsychopharmacology 26: 479–488. 11927172 10.1016/S0893-133X(01)00367-0

[7] Ridenour TA, Maldonado-Molina M, Compton WM, Spitznagel EL, Cottler LB (2005) Factors associated with the transition from abuse to dependence among substance abusers: implications for a measure of addictive liability. Drug Alcohol Depend 80: 1–14. 16157227 10.1016/j.drugalcdep.2005.02.005 PMC1435339

[8] Deroche-Gamonet V, Belin D, Piazza PV (2004) Evidence for addiction-like behavior in the rat. Science 305: 1014–1017. 15310906 10.1126/science.1099020

[9] Kasanetz F, Deroche-Gamonet V, Berson N, Balado E, Lafourcade M, et al (2010) Transition to addiction is associated with a persistent impairment in synaptic plasticity. Science 328: 1709–1712. 20576893 10.1126/science.1187801

[10] Lopez-Quintero C, Perez de los Cobos J, Hasin DS, Okuda M, Wang S, et al (2011) Probability and predictors of transition from first use to dependence on nicotine, alcohol, cannabis, and cocaine: results of the National Epidemiologic Survey on Alcohol and Related Conditions (NESARC). Drug Alcohol Depend 115: 120–130. 21145178 10.1016/j.drugalcdep.2010.11.004 PMC3069146

[11] Vink JM, Willemsen G, Boomsma DI (2005) Heritability of smoking initiation and nicotine dependence. Behav Genet 35: 397–406. 15971021 10.1007/s10519-004-1327-8

[12] Koob GF (2008) Hedonic Homeostatic Dysregulation as a Driver of Drug-Seeking Behavior. Drug Discov Today Dis Models 5: 207–215. 20054425 10.1016/j.ddmod.2009.04.002 PMC2801885

[13] Zhang D, Shao C, Shao M, Yan P, Wang Y, et al (2007) Effect of mu-opioid receptor gene polymorphisms on heroin-induced subjective responses in a Chinese population. Biol Psychiatry 61: 1244–1251. 17157823 10.1016/j.biopsych.2006.07.012

[14] Lett BT (1989) Repeated exposures intensify rather than diminish the rewarding effects of amphetamine, morphine, and cocaine. Psychopharmacology (Berl) 98: 357–362. 2546170 10.1007/BF00451687

[15] Manzanedo C, Aguilar MA, Rodriguez-Arias M, Minarro J (2005) Sensitization to the rewarding effects of morphine depends on dopamine. Neuroreport 16: 201–205. 15671878 10.1097/00001756-200502080-00028

[16] Koob GF, Le Moal M (2001) Drug addiction, dysregulation of reward, and allostasis. Neuropsychopharmacology 24: 97–129. 11120394 10.1016/S0893-133X(00)00195-0

[17] Wise RA, Rompre PP (1989) Brain dopamine and reward. Annu Rev Psychol 40: 191–225. 2648975 10.1146/annurev.ps.40.020189.001203

[18] Yacubian J, Buchel C (2009) The genetic basis of individual differences in reward processing and the link to addictive behavior and social cognition. Neuroscience 164: 55–71. 19446009 10.1016/j.neuroscience.2009.05.015

[19] Jacobs MM, Okvist A, Horvath M, Keller E, Bannon MJ, et al (2012) Dopamine receptor D1 and postsynaptic density gene variants associate with opiate abuse and striatal expression levels. Mol Psychiatry 10.1038/mp.2012.140 PMC3637428 23044706

[20] Huang W, Ma JZ, Payne TJ, Beuten J, Dupont RT, et al (2008) Significant association of DRD1 with nicotine dependence. Hum Genet 123: 133–140. 18092181 10.1007/s00439-007-0453-9

[21] Kim DJ, Park BL, Yoon S, Lee HK, Joe KH, et al (2007) 5′ UTR polymorphism of dopamine receptor D1 (DRD1) associated with severity and temperament of alcoholism. Biochem Biophys Res Commun 357: 1135–1141. 17466946 10.1016/j.bbrc.2007.04.074

[22] da Silva Lobo DS, Vallada HP, Knight J, Martins SS, Tavares H, et al (2007) Dopamine genes and pathological gambling in discordant sib-pairs. J Gambl Stud 23: 421–433. 17394052 10.1007/s10899-007-9060-x

[23] Zhu F, Yan CX, Wang Q, Zhu YS, Zhao Y, et al (2011) An association study between dopamine D1 receptor gene polymorphisms and the risk of schizophrenia. Brain Res 1420: 106–113. 21955727 10.1016/j.brainres.2011.08.069

[24] Hettinger JA, Liu X, Schwartz CE, Michaelis RC, Holden JJ (2008) A DRD1 haplotype is associated with risk for autism spectrum disorders in male-only affected sib-pair families. Am J Med Genet B Neuropsychiatr Genet 147B: 628–636. 18205172 10.1002/ajmg.b.30655

[25] Huang W, Li MD (2009) Differential allelic expression of dopamine D1 receptor gene (DRD1) is modulated by microRNA miR-504. Biol Psychiatry 65: 702–705. 19135651 10.1016/j.biopsych.2008.11.024 PMC2678413

[26] Jurinke C, van den Boom D, Cantor CR, Koster H (2002) Automated genotyping using the DNA MassArray technology. Methods Mol Biol 187: 179–192. 12013745 10.1385/1-59259-273-2:179

[27] Dupont WD, Plummer WD Jr (1998) Power and sample size calculations for studies involving linear regression. Control Clin Trials 19: 589–601. 9875838 10.1016/s0197-2456(98)00037-3

[28] Barrett JC (2009) Haploview: Visualization and analysis of SNP genotype data. Cold Spring Harb Protoc 2009: pdb ip71. 20147036 10.1101/pdb.ip71

[29] Kreek MJ, Nielsen DA, Butelman ER, LaForge KS (2005) Genetic influences on impulsivity, risk taking, stress responsivity and vulnerability to drug abuse and addiction. Nat Neurosci 8: 1450–1457. 16251987 10.1038/nn1583

[30] Depue RA, Collins PF (1999) Neurobiology of the structure of personality: dopamine, facilitation of incentive motivation, and extraversion. Behav Brain Sci 22: 491–517 discussion 518–469. 11301519 10.1017/s0140525x99002046

[31] Wise RA (2000) Addiction becomes a brain disease. Neuron 26: 27–33. 10798389 10.1016/s0896-6273(00)81134-4

[32] Piazza PV, Deroche-Gamonent V, Rouge-Pont F, Le Moal M (2000) Vertical shifts in self-administration dose-response functions predict a drug-vulnerable phenotype predisposed to addiction. J Neurosci 20: 4226–4232. 10818158 10.1523/JNEUROSCI.20-11-04226.2000 PMC6772616

[33] Caine SB, Thomsen M, Gabriel KI, Berkowitz JS, Gold LH, et al (2007) Lack of self-administration of cocaine in dopamine D1 receptor knock-out mice. J Neurosci 27: 13140–13150. 18045908 10.1523/JNEUROSCI.2284-07.2007 PMC2747091

[34] Gerrits MA, Ramsey NF, Wolterink G, van Ree JM (1994) Lack of evidence for an involvement of nucleus accumbens dopamine D1 receptors in the initiation of heroin self-administration in the rat. Psychopharmacology (Berl) 114: 486–494. 7855207 10.1007/BF02249340

[35] Glick SD, Cox RD (1975) Dopaminergic and cholinergic influences on morphine self-administration in rats. Res Commun Chem Pathol Pharmacol 12: 17–24. 1188183

[36] Rowlett JK, Platt DM, Yao WD, Spealman RD (2007) Modulation of heroin and cocaine self-administration by dopamine D1- and D2-like receptor agonists in rhesus monkeys. J Pharmacol Exp Ther 321: 1135–1143. 17351103 10.1124/jpet.107.120766

[37] Liu J, Nickolenko J, Sharp FR (1994) Morphine induces c-fos and junB in striatum and nucleus accumbens via D1 and N-methyl-D-aspartate receptors. Proc Natl Acad Sci U S A 91: 8537–8541. 8078918 10.1073/pnas.91.18.8537 PMC44641

[38] Muller DL, Unterwald EM (2005) D1 dopamine receptors modulate deltaFosB induction in rat striatum after intermittent morphine administration. J Pharmacol Exp Ther 314: 148–154. 15772255 10.1124/jpet.105.083410

[39] Zachariou V, Bolanos CA, Selley DE, Theobald D, Cassidy MP, et al (2006) An essential role for DeltaFosB in the nucleus accumbens in morphine action. Nat Neurosci 9: 205–211. 16415864 10.1038/nn1636

[40] Borgkvist A, Valjent E, Santini E, Herve D, Girault JA, et al (2008) Delayed, context- and dopamine D1 receptor-dependent activation of ERK in morphine-sensitized mice. Neuropharmacology 55: 230–237. 18614186 10.1016/j.neuropharm.2008.05.028

[41] Dudman JT, Eaton ME, Rajadhyaksha A, Macias W, Taher M, et al (2003) Dopamine D1 receptors mediate CREB phosphorylation via phosphorylation of the NMDA receptor at Ser897-NR1. J Neurochem 87: 922–934. 14622123 10.1046/j.1471-4159.2003.02067.x PMC4203348

[42] Nestler EJ (2004) Molecular mechanisms of drug addiction. Neuropharmacology 47 Suppl 1: 24–32. 15464123 10.1016/j.neuropharm.2004.06.031

[43] Kauer JA, Malenka RC (2007) Synaptic plasticity and addiction. Nat Rev Neurosci 8: 844–858. 17948030 10.1038/nrn2234

[44] Wise RA, Leone P, Rivest R, Leeb K (1995) Elevations of nucleus accumbens dopamine and DOPAC levels during intravenous heroin self-administration. Synapse 21: 140–148. 8584975 10.1002/syn.890210207

[45] Hilario MR, Turner JR, Blendy JA (2012) Reward sensitization: effects of repeated nicotine exposure and withdrawal in mice. Neuropsychopharmacology 37: 2661–2670. 22828747 10.1038/npp.2012.130 PMC3473332

[46] Bie B, Wang Y, Cai YQ, Zhang Z, Hou YY, et al (2012) Upregulation of Nerve Growth Factor in Central Amygdala Increases Sensitivity to Opioid Reward. Neuropsychopharmacology 10.1038/npp.2012.144 PMC3499709 22871918

[47] Kourrich S, Klug JR, Mayford M, Thomas MJ (2012) AMPAR-independent effect of striatal alphaCaMKII promotes the sensitization of cocaine reward. J Neurosci 32: 6578–6586. 22573680 10.1523/JNEUROSCI.6391-11.2012 PMC3448780

[48] Lintas A, Chi N, Lauzon NM, Bishop SF, Gholizadeh S, et al (2011) Identification of a dopamine receptor-mediated opiate reward memory switch in the basolateral amygdala-nucleus accumbens circuit. J Neurosci 31: 11172–11183. 21813678 10.1523/JNEUROSCI.1781-11.2011 PMC6623365

[49] Bahi A, Dreyer JL (2012) Involvement of nucleus accumbens dopamine D1 receptors in ethanol drinking, ethanol-induced conditioned place preference, and ethanol-induced psychomotor sensitization in mice. Psychopharmacology (Berl) 222: 141–153. 22222864 10.1007/s00213-011-2630-8

[50] Zhang J, Wang J, Wu Y (2012) An improved approach for accurate and efficient calling of structural variations with low-coverage sequence data. BMC Bioinformatics 13 Suppl 6: S6. 10.1186/1471-2105-13-S6-S6 PMC3358659 22537045

