# Supplementary figures and images for: Dopamine D1 Receptor Gene Variation Modulates Opioid Dependence Risk by Affecting Transition to Addiction
Source: PLoS One. 2013 Aug 16;8(8):e70805. doi: 10.1371/journal.pone.0070805 (PMC3745389; doi:10.1371/journal.pone.0070805)

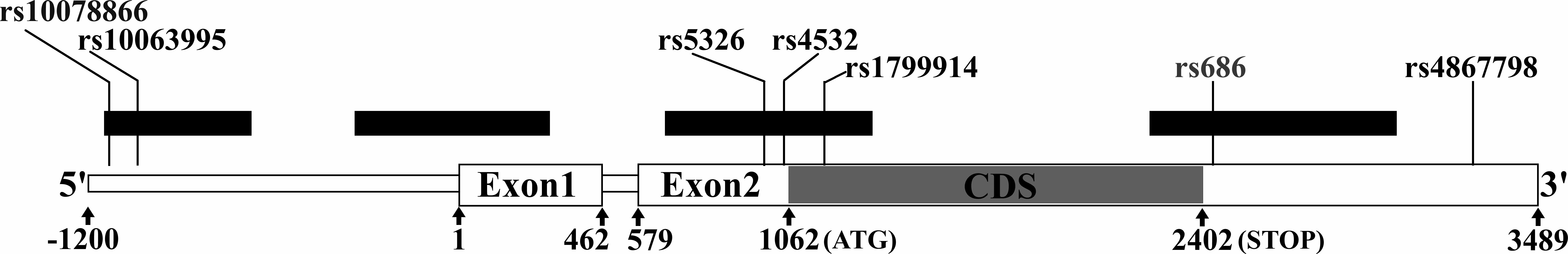

Supplement: Figure S1 — Gene structure of human DRD1 , showing the re-sequencing fragments and the relative positions of the 7 SNPs used in our study. The black squares above the chart of gene structure indicate the fragments we targeted for re-sequencing. (TIF) [file pone.0070805.s001.tif]

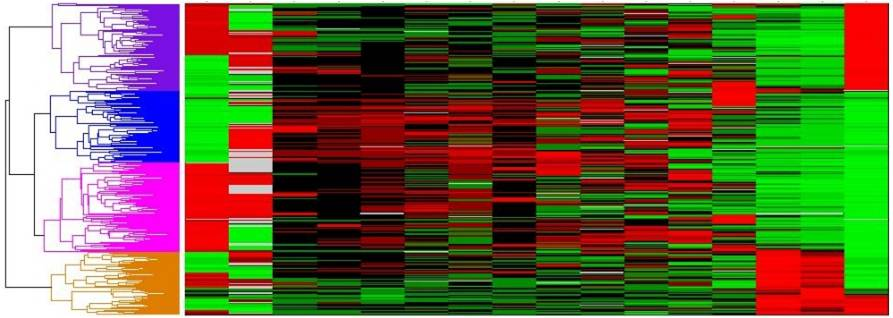

Supplement: Figure S2 — Four subgroups of addicts defined by a multivariate cluster analysis. The columns with different color indicate the different clinical measures that characterized opioid use and related behaviors, mainly including the age of onset, gender, type of opioid, administration route, daily administration dosage of opioid before methadone treatment, use times of opioid daily before methadone treatment, DTFUD, subjective response, and so on. (TIF) [file pone.0070805.s002.tif]
